# Supplementary material for: L-DOS47 Elevates Pancreatic Cancer Tumor pH and Enhances Response to Immunotherapy
Source: Biomedicines. 2024 Feb 19;12(2):461. doi: 10.3390/biomedicines12020461 (PMC10886509; doi:10.3390/biomedicines12020461)
Supplement: Supplementary file 1 [file biomedicines-12-00461-s001.zip › biomedicines-2800211-supplementary.pdf]

Supplemental Figures

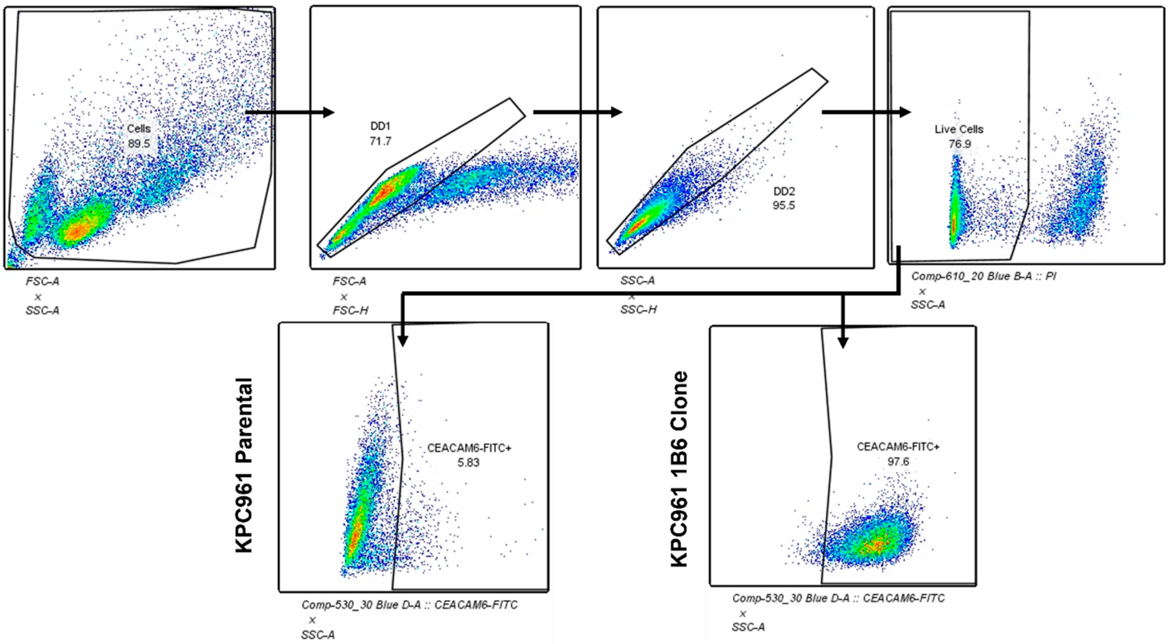

Supplemental Fig S1. Gating strategy used for flow cytometer analysis. FACS plots are shown as representative example of the gating strategy used to quantify the CEACAM6 expression in KPC961 clone 1B6 cells after transduction of human CEACAM6.

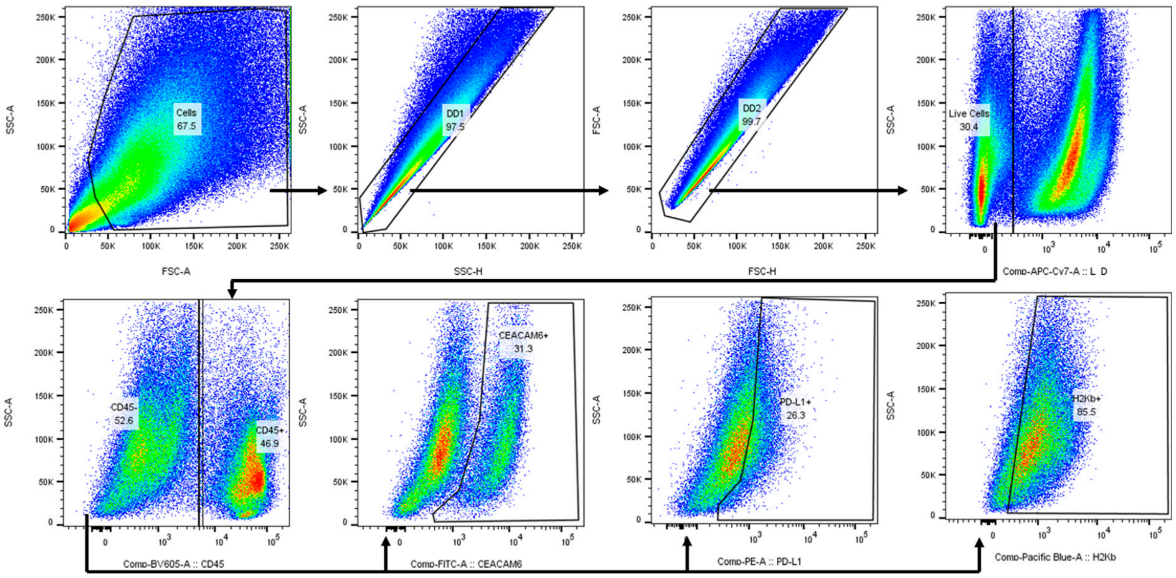

Supplemental Fig S2. Gating strategy used for flow cytometer analysis. FACS plots are shown as representative example of the gating strategy used to quantify the expression of CEACAM6, PDL-1, and H2Kb in inoculated KPC961-1B6 tumors

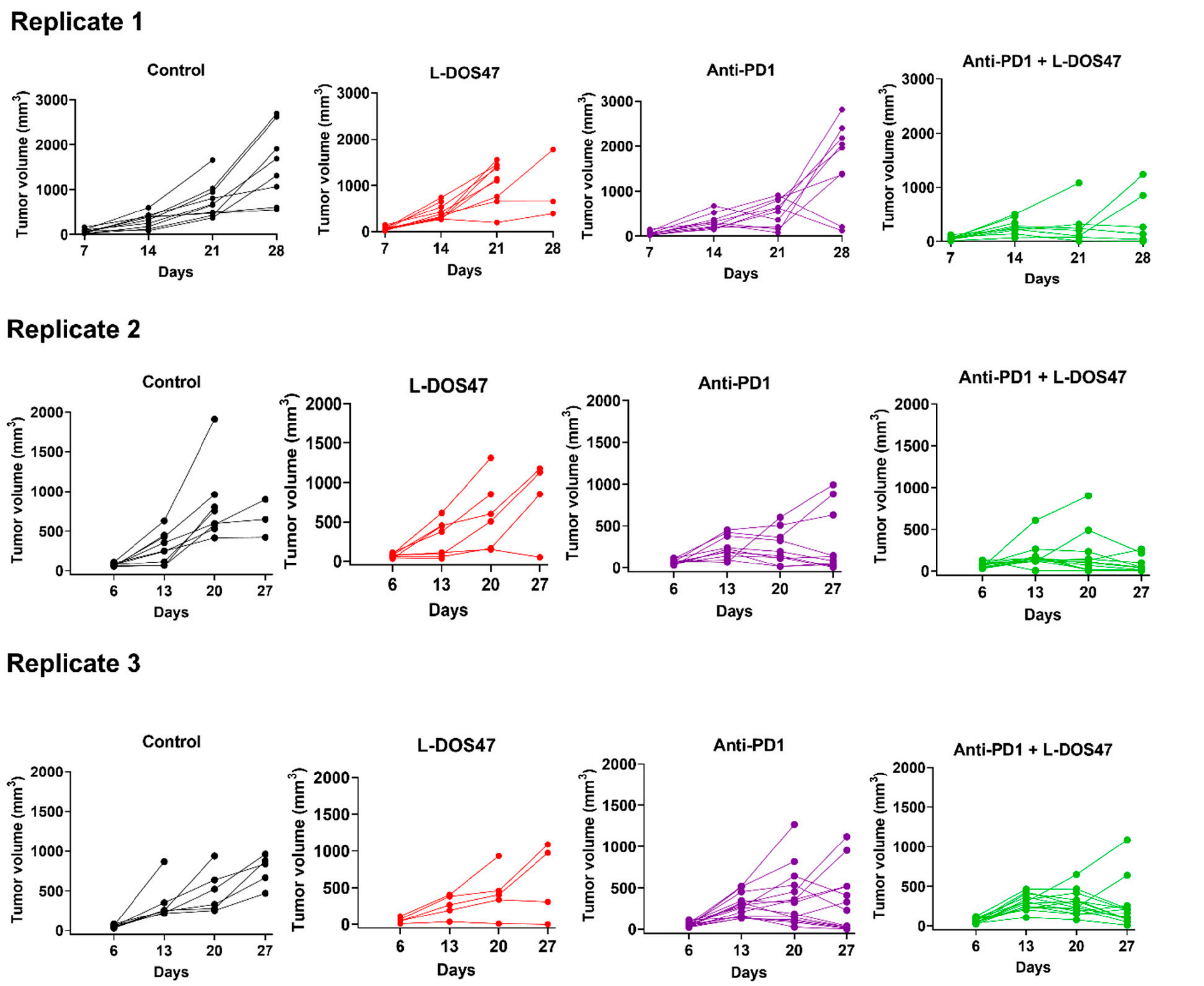

Supplemental Fig S3. **Individual tumor growth for each therapy group for replicates 1, 2 and 3.** Number of mice per group in each replicates were: Replicate 1 Control=10, anti-PD1=10, L-DOS47 = 10, anti-PD1 + L-DOS47 =9; Replicate 2 Control = 9, anti-PD1=10, L-DOS47 =8, anti-PD1 + L-DOS47 =12; and replicate 3 Control =7, anti-PD1 =15, L-DOS47 =5, anti-PD1 + L-DOS47 =14.

Supplemental Tables

Table S1. Comparison of cumulative pHe distribution before and after L-DOS47 for each post-L-DOS47 time point group, stratified by pHe categories (pHe ≤ 6.6 and pHe > 6.6).

**4 hours post-L-DOS47**				
**Category**	**Group**	**Mean**	**SD**	**p-value**
pHe ≤ 6.6	Pre-L-DOS47	6.54	0.33	<0.001
	Post-L-DOS47	6.67	0.39	
pHe > 6.6	Pre-L-DOS47	6.76	0.29	<0.001
	Post-L-DOS47	6.65	0.35	
**18 hours post-L-DOS47**				
**Category**	**Group**	**Mean**	**SD**	**p-value**
pHe ≤ 6.6	Pre-L-DOS47	6.56	0.29	0.51
	Post-L-DOS47	6.57	0.33	
pHe > 6.6	Pre-L-DOS47	6.67	0.29	<0.001
	Post-L-DOS47	6.54	0.33	
**24 hours post-L-DOS47**				
**Category**	**Group**	**Mean**	**SD**	**p-value**
pHe ≤ 6.6	Pre-L-DOS47	6.53	0.36	<0.001
	Post-L-DOS47	6.64	0.35	
pHe > 6.6	Pre-L-DOS47	6.69	0.32	<0.001
	Post-L-DOS47	6.60	0.38	
**48 hours post-L-DOS47**				
**Category**	**Group**	**Mean**	**SD**	**p-value**
pHe ≤ 6.6	Pre-L-DOS47	6.53	0.33	<0.001
	Post-L-DOS47	6.60	0.38	
pHe > 6.6	Pre-L-DOS47	6.73	0.35	0.03
	Post-L-DOS47	6.70	0.36	
**72 hours post-L-DOS47**				
**Category**	**Group**	**Mean**	**SD**	**p-value**
pHe ≤ 6.6	Pre-L-DOS47	6.54	0.30	<0.001
	Post-L-DOS47	6.65	0.40	
pHe > 6.6	Pre-L-DOS47	6.75	0.34	<0.001
	Post-L-DOS47	6.65	0.38	
**96 hours post-L-DOS47**				
**Category**	**Group**	**Mean**	**SD**	**p-value**
pHe ≤ 6.6	Pre-L-DOS47	6.53	0.41	0.01
	Post-L-DOS47	6.65	0.37	
pHe > 6.6	Pre-L-DOS47	6.68	0.36	<0.001
	Post-L-DOS47	6.59	0.34	

Table S2. Coefficients from the linear mixed effects model for tumor growth by treatment arm for Experiments 1, 2 and 3 combined. The reference treatment arm (represented by the intercept) is Anti-PD1 + L-DOS47.

Fixed effects	Estimate	Std. Error	df	t value	Pr(>|t|)
(Intercept)	6.43	0.30	474.58	21.06	< 2e-16
Anti-PD1	-0.36	0.42	469.39	-0.86	0.38
L-DOS47	-0.88	0.47	462.44	-1.85	0.06
Control	-1.12	0.46	468.34	-2.42	0.01
day	0.02	0.01	413.99	1.51	0.13
Anti-PD1: day	0.04	0.02	414.16	2.17	0.02
LDOS47: day	0.11	0.02	414.01	4.77	2.50e-06
Control: day	0.14	0.02	414.16	5.96	5.18e-09
